# A Urine Metabonomics Study of Rat Bladder Cancer by Combining Gas Chromatography-Mass Spectrometry with Random Forest Algorithm

**DOI:** 10.1155/2020/8839215

**Published:** 2020-09-21

**Authors:** Mengchan Fang, Fan Liu, Lingling Huang, Liqing Wu, Lan Guo, Yiqun Wan

**Affiliations:** ^1^College of Chemistry, Nanchang University, Nanchang 330031, China; ^2^Jiangxi Province Key Laboratory of Modern Analytical Science, Nanchang University, Nanchang 330031, China; ^3^Department of Pathology, 3rd Affiliated Hospital of Nanchang University, Nanchang 330008, China

## Abstract

A urine metabolomics study based on gas chromatography-mass spectrometry (GC-MS) and multivariate statistical analysis was applied to distinguish rat bladder cancer. Urine samples with different stages were collected from animal models, i.e., the early stage, medium stage, and advanced stage of the bladder cancer model group and healthy group. After resolving urea with urease, the urine samples were extracted with methanol and, then, derived with N, O-Bis(trimethylsilyl) trifluoroacetamide and trimethylchlorosilane (BSTFA + TMCS, 99 : 1, v/v), before analyzed by GC-MS. Three classification models, i.e., healthy control vs. early- and middle-stage groups, healthy control vs. advanced-stage group, and early- and middle-stage groups vs. advanced-stage group, were established to analyze these experimental data by using Random Forests (RF) algorithm, respectively. The classification results showed that combining random forest algorithm with metabolites characters, the differences caused by the progress of disease could be effectively exhibited. Our results showed that glyceric acid, 2, 3-dihydroxybutanoic acid, N-(oxohexyl)-glycine, and D-turanose had higher contributions in classification of different groups. The pathway analysis results showed that these metabolites had relationships with starch and sucrose, glycine, serine, threonine, and galactose metabolism. Our study results suggested that urine metabolomics was an effective approach for disease diagnosis.

## 1. Introduction

Bladder cancer (BC) is a common malignant tumor disease of the urinary tract, and its incidence and mortality have always occupied the first place in the urinary reproductive system tumors. Due to the easy relapse characteristic, BC has been the focus of researchers to search tumor markers for the early diagnosis and postoperative evaluation to improve the survival rate of bladder cancer patients [1].

Metabonomics has been widely used in the research of diseases diagnosis [2], pharmacological [3] and toxicological mechanisms [4], plant and microorganism metabolism [5, 6], and so on [7]. Two analysis methods were widely used in metabonomics. One is the metabonomics technology basing on nuclear magnetic resonance (NMR) [8–10], and the other is chromatography-mass spectrometry [11–13]. NMR technology has the advantages of fast analysis and simple sample preparation, while it also possesses the disadvantage of low sensitivity [14]. Chromatography-mass spectrometry technology mainly contains gas chromatography-mass spectrometry (GC-MS) [15, 16], liquid chromatography-mass spectrometry (LC-MS) [17–19], and capillary electrophoresis-mass spectrometry (CE-MS) [20–22]. Among these technologies, GC-MS has been widely used in metabonomics studies owing to its high sensitivity, strong analysis ability, and possessing more mature commercial mass spectrum library [23]. The samples used in metabonomics commonly are biological fluids, such as urine [24, 25], serum [26, 27], interstitial fluid [28], and cerebrospinal fluid [29]. Due to the characteristic of weak volatility and poor thermal stability, many analytes in biological fluids, such as amino acids and organic acids, must be derivatized before GC-MS analysis. The development of modern derivatization technology has greatly promoted the application of GC-MS in metabonomics [30, 31].

Some studies were proposed to discover biomarkers for BC [32–36] in recent years. Pasikanti et al. [37] have developed a noninvasive method for the diagnosis and surveillance of BC progression by using the GC × GC-MS method. Peng et al. [38] reported a chemical isotope labeling LC-MS metabolomics method based on the universal metabolome-standard method with low CV for all quantified metabolites. This method was used to screen potential biomarkers from urine samples for bladder cancer diagnosis. However, the current study for diagnosis of BC is mostly focusing on high-grade tumors. Discovering biomarkers which could characterize different stages of BC is of more useful in diagnosis and prognostics of bladder cancer [39]. Alberice et al. [40] followed up 48 urothelial bladder cancer patients by using urine metabolomics, and 27 metabolites were highlighted between different BC stages and recurrence. However, the BC patients following up is difficult, especially the early-stage BC samples collection is of a hard work since most BC patients are in high stage when diagnosed.

Therefore, in this study, the rat bladder cancer model was established, and the urine metabonomics was studied with GC-MS technology. The rat urine samples of the advanced stage, medium stage, and early stage of the bladder cancer model group and healthy group had been detected to establish the bladder cancer urine metabolic fingerprint, and then, the experimental data were analyzed with algorithm of random forests [41, 42] and used for the preliminary exploration of the tumor markers of BC.

## 2. Experiment

### 2.1. Standards and Reagents

Twenty amino acid and nine carbohydrate standards, including isoleucine, lysine hydrochloride, cysteine, glycine, tryptophan, glutamic acid, valine, threonine, proline, glutamate, leucine, methionine, phenylalanine, cystine, aspartic acid, asparagine, histidine, serine, 4-hydroxy proline, arginine, galactose, fructose, sucrose, glucose, mannose, ribose, xylose, arabinose, and glucuronic acid, were purchased from Sigma-Aldrich (St. Louis, MO, USA). Urease was of analytical grade and purchased from Sigma-Aldrich (St. Louis, MO, USA). Methanol, acetonitrile, and acetone were of chromatographical grade and purchased from Merck Company (Darmstadt, Germany); n-docosane, pyridine, and n-heptane were of analytical grade and purchased from Sinopharm Chemical Reagent Co. Ltd. (Shanghai, China). N,O-Bis(trimethylsilyl) trifluoroacetamide + trimethylchlorosilane (BSTFA + TMCS, 99 : 1, v/v) and methoxyamine hydrochloride were purchased from Supelco (Bellefonte, PA, USA).

### 2.2. Rat Bladder Cancer Model

All animal experiments were conducted according to the institutional guidelines of Medical College of Nanchang University. One hundred and eighty male SD rats (6 weeks old) weighing 150–160 g were purchased from Hunan Experimental Animal Co. LTD. (Hunan, China), with the license number of SCXK (Hunan) 2009–0004 and the qualified number of HNASLKJ20102985. The rats were randomly assigned to two groups, forty-five rats were in the control group and one hundred and thirty-five rats were in the model group. Bladder tumors were induced by adding 0.05% BBN (N-butyl-N-(4-hydroxybutyl) nitrosamine) (Tokyo, Japan) to freely available drinking water after a week of adaptive period in the experimental animal room. BBN was continuously administered for 35 weeks in the experiment.

### 2.3. Histologic Examination

After induced by BBN, three rats of the BC model group were randomly selected for histological examination every five weeks. The rats were anesthetized by intraperitoneal injection of ketamine (0.6 mL/50 g), and the rats were killed under deep anesthesia. After death, rats were catheterized and 0.2 mL buffered formalin was instilled into the bladder. The urethras were, then, ligated, and the bladders were removed. Fixed specimens were embedded in paraffin, and 4–5 *μ*m thick horizontal slices in each rat were prepared at 2 mm intervals followed by routine hematoxylin and eosin staining. Then, the prepared pathological sections were detected with optical microscopy using ordinary white light by the same pathologists.

### 2.4. Sample Preparation

The rat urine samples of the control group and model group were collected with a metabolism cage for 24 h every five weeks. The urine samples were centrifuged immediately for 20 min at 4000 r/min to remove protein with a TDL-5-A low-speed tabletop centrifuge (Shanghai Anting Scientific Instrument Factory, China). Then, 150 *μ*L supernatant liquid was mixed with 100 *μ*L of urease solution (2 mg/mL) in a 1 mL centrifuge tube and heated at 37°C in a DGG-9140BD constant temperature oven (Shanghai Senxin Experimental Instrument Co., Ltd., China) for 30 min to decompose urea. After adding 800 *μ*L methanol, the mixture was homogenized for 1 min at 1800 r/min with a MS2 mini shaker (Guangzhou Yike Lab Technology LTM Co., China), followed by ultrasonic processing in an ice bath for 10 min with a KQ-100DE ultrasonic cleaner (Kunshan Ultrasonic Instruments Co., Ltd., China) and centrifuging for 10 min at 12000 r/min with a TDL-16G high-speed tabletop centrifuge (Shanghai Anting Scientific Instrument Factory, China). Then, 500 *μ*l supernatant liquid was transferred into a 2 mL centrifuge tube and evaporated to dryness under a gentle nitrogen stream, and then, 75 *μ*L methoxyamine hydrochloride solution (20 mg/mL in pyridine) was added into the tube to react for 1 h at 70°C. After the reaction, the mixture was cooled to room temperature and reacted with 75 *μ*l BSTFA + TMCS (99 : 1, v/v) for 1 h at room temperature to form trimethylsilyl (TMS) derivatives. Finally, the reaction was terminated by the addition of 150 *μ*L n-heptane (containing 0.1 g/L n-docosane, which was used as an internal standard substance), and the products of the derivative reaction were analyzed by GC-MS.

### 2.5. GC-MS Analysis

GC-MS analysis was carried out using an Agilent 6890N Gas Chromatograph (Agilent Technologies, Palo Alto, CA, USA) integrated with an Agilent 7683 series autosampler and a 5973 I mass selective detector (MSD). The analytes were separated on a 30 m × 0.25 mm i.d. × 0.25 *μ*m film thickness DB-5MS fused-silica capillary column (Agilent Technologies). The injector was set at 270°C, and the carrier gas was UHP helium at a flow rate of 1.0 mL/min. The samples were injected in a splitless mode. The oven temperature was initially at 70°C for 5 min; increased at a rate of 20°C/min up to 160°C, held for 4 min; increased at a rate of 10°C/min up to 300°C; and held for 1.5 min. The ion source, quadrupole, and transfer line temperature were set at 230, 150°C, and 280°C, respectively. The detector was operated at 70 ev in an electron impact (EI) mode with full scan (60∼600 amu). The solvent delay and injection volume were set as 5 min and 2 *μ*L, respectively. All the data was collected and analyzed with MSD ChemStation D.01.02 software (Agilent Technologies), with the NIST02 mass database.

## 3. Results and Discussion

### 3.1. Optimization of the Urine Sample Preparation Method

#### 3.1.1. Selection of Extraction Solvent

There are many kinds of endogenous metabolites with high polarity difference and wide concentration range in rat urine. To obtain more information of endogenous metabolites, the extraction efficiencies of different organic solvents were investigated. It was found that methanol and acetonitrile performed better than others. Finally, methanol was chosen as the extraction solvent in this study because of the lower toxicity of methanol than acetonitrile.

#### 3.1.2. Optimization of the Urea Decomposition Conditions

The urine should be treated with urease to decompose the urea before GC-MS analysis, since the urea with high concentration in urine may affect other compounds analysis. Effects of several factors including the dosage of urease, decomposition temperature, and decomposition time were investigated. 50 *µ*L, 100 *µ*L, and 200 *µ*L urease solution (2 mg/mL) was added to a 150 *µ*L urine sample and reacted at 60°C, 37°C, and 20°C for 15 min, 30 min, and 60 min. Then, its decomposition effect was examined. The results are listed in Supplementary Materials. Finally, the optimum conditions for urea decomposing in the 150 *μ*L urine sample were set as dosage of urease solution (2 mg/mL), 100 *μ*L; decomposition temperature, 37°C; and decomposition time, 30 min.

#### 3.1.3. Optimization of the Multistep Temperature Program

**Figure 1:**
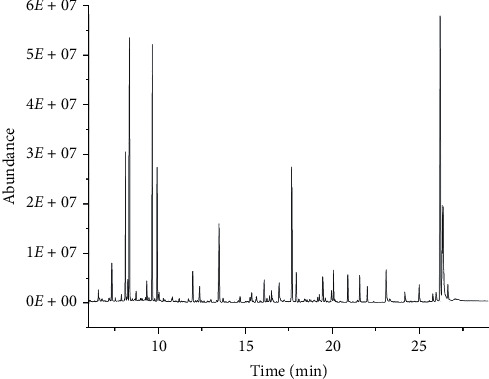
The total ion chromatograms (TICs) of rat bladder cancer urine sample.

To obtain better separation and get more information of endogenous metabolites, four multistep temperature programs as shown in below were carried out on a DB-5MS capillary column in this study. Considering the number of chromatographic peaks and their separation efficiency, the third temperature-rising program was selected as the experimental condition. The total ion chromatogram (TIC) of the actual sample is shown in Figure 1, and there were more than 40 peaks obtained in 29 min with good separation efficiency.

1. The temperature was initially at 85°C for 5 min, increased at a rate of 10°C/min up to 300°C, and held for 10 min
2. The temperature was initially at 100°C for 3 min, increased at a rate of 8°C/min, and held for 2 min
3. The temperature was initially at 70°C for 5 min, increased at a rate of 20°C/min up to 160°C, held for 4 min, increased at a rate of 10°C/min up to 300°C, and held for 1.5 min
4. The temperature was initially at 85°C for 5 min, increased at a rate of 8°C/min up to 205°C, held for 5 min, increased at a rate of 8°C/min up to 300°C, and held for 5 min

### 3.2. Urine Sample Analysis

**Figure 2:**
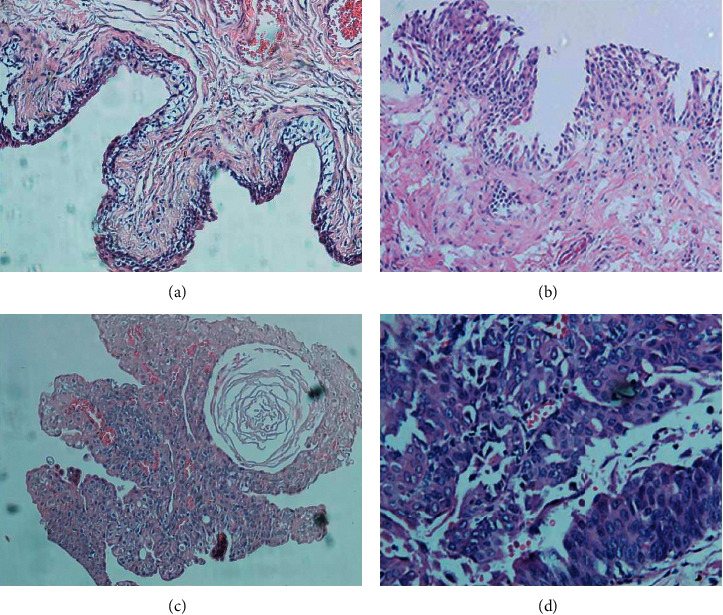
Histopathological specimen examination of the rat bladder of the control (a), 15th week (b), 25th week (c), and 35th week (d). *Note.* (a) The epithelial tissue was composed of 2–3 layers, and the cells were extremely obvious, without abnormality, and arranged in an orderly manner. (b)There were papillary hyperplasia in the partial region, the epithelial cell had 4–6 layers, polarity had a little disorder, and the cell's morphology and size had a certain atypia. (c) The layer number of tumor cell increased significantly with ball-shaped distribution, the sizes of tumor cells were different, the nucleus was deeply dyed and showed polymorphism, the atypia was obvious, and some tumor cells showed the characteristics of squamous cell tumor differentiation. (d) The nucleus was deeply stained, the nuclear membrane was thickened, the nucleoli were obvious and showed pathological karyokinesis, and the muscularis was deeply infiltrated.

The different stages of bladder cancer rats were confirmed by the histopathology analysis. Because the rat bladder of fifteenth, twenty-fifth, and thirty-fifth week after BBN inducing displayed the typical characteristics of early, medium, and advanced stage tumors (shown in Figure 2), the urine samples collected in these three weeks were chosen as early-, medium-, and advanced-stage samples, respectively. The early, medium, and advanced stage of the bladder cancer model group and healthy group include 45 samples each, and all the samples were analyzed by GC-MS under the optimized conditions with 41 common peaks obtained. To identify the complex metabolites in urine, the metabolites were divided into three categories: amino acids, carbohydrates, and fatty acids. Because the NIST database has rich information of fatty acid derivatives but poor information of amino acid derivatives and carbohydrate derivatives, the fatty acid derivatives were identified directly with the NIST database, while the amino acids and carbohydrate derivatives were identified by standard compounds. The quantitative results of metabolites were given as the peak area ratio of the analyte to the internal standard of n-docosane. Finally, all the 41 common peaks were identified successfully, and the qualitative and quantitative results are listed in Table 1.

### 3.3. Experimental Data Analysis

**Figure 3:**
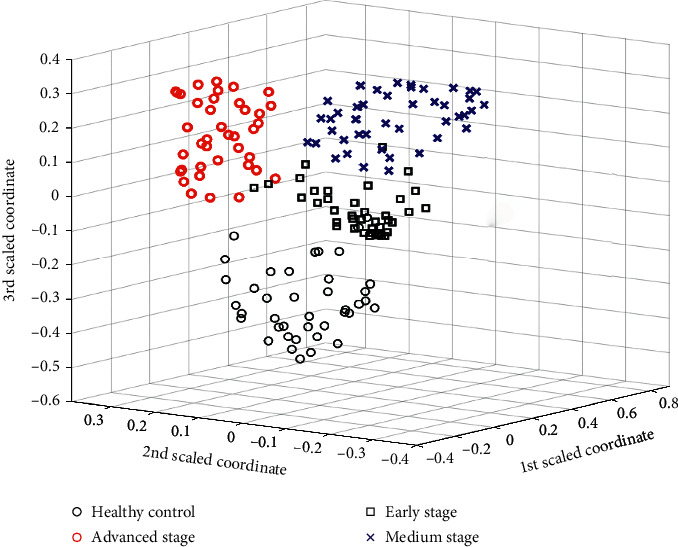
Analysis results of four group samples with random forest.

The classifiers commonly used in metabolomics include partial least squares discriminant analysis (PLS-DA), support vector machine (SVM), and random forest (RF) [43]. PLS-DA can reduce the impact of multiple correlations between variables, but it is easy to overfit the data, and the selected biomarkers are not robust enough. SVM can solve small sample classification, high-dimensional data classification, and nonlinear problems; however, it is more difficult to train large-scale samples and deal with multiclassification problems. Random forests (RF) algorithm was first proposed by Breiman in 2001 [44] and widely used [45, 46] since it can distinguish the differences between different group samples effectively. RF is a supervised machine learning classifier, including a collection of classification and regression trees. It consists of many different decision trees, which are grown based on various guide samples. Each tree voted for the sample for classification, and RF chose the majority vote to determine the final classification result. It has good performance and has great advantages compared with other algorithms. RF can handle high-dimensional data (many feature data) and does not need to make feature selection. After training, random forest can screen out more important features. Compared with other classification models, its biased estimation of classification results is low, which makes random forests applicable to many research fields. RF was adopted to classify metabolites among the four groups of rat urines, and the obtained multidimensional scaling (MDS) figure is shown in Figure 3. As shown in Figure 3, the differences among different groups were obviously in the classification plot. The cancer groups could be effectively distinguished from the healthy groups; moreover, the advanced-stage groups could also be distinguished from the early and middle-stage groups. However, the sample points of the early stage and middle stage are somewhat overlapped. The results suggested that the metabolic pathways of rat bladder cancer were obviously different from that of healthy rats, which were similar in the early and middle stage, but in the advanced stage, the metabolic pathways had changed significantly due to tumor deterioration and excessive nutrient consumption.

Thus, we further established three classification models, i.e., healthy control vs. early- and middle-stage groups, healthy control vs. advanced-stage group, and early- and middle-stage groups vs. advanced stage group, respectively. The classification results for three models are listed in Table 2. It is obvious that the healthy control vs. advanced-stage group had the best classification accuracy, indicating the signification differences of metabolic features between the healthy control and advanced-stage group; and the healthy control vs. early- and middle-stage groups also had a classification accuracy of 96.06%. These results suggested that the proposed metabolomics approach can reflect the differences among different groups, with the progress of the disease.

**Figure 4:**
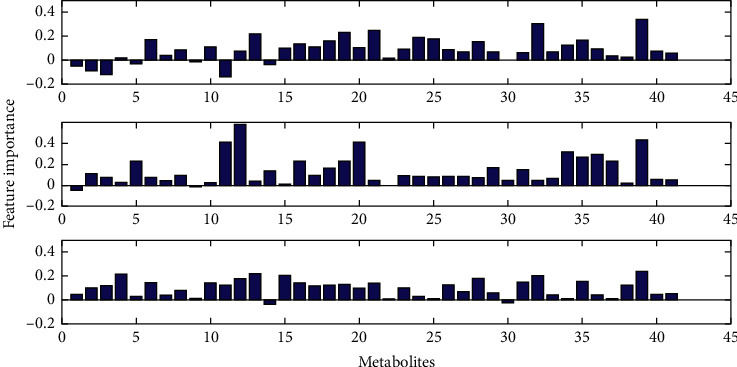
The variable importance of metabolites. (a) Healthy control vs. early- and middle-stage groups, (b) healthy control vs. advanced-stage group, and (c) early- and middle-stage groups vs. advanced stage group.

During the classification model establishing, the importance of the metabolites was calculated. The metabolites with higher importance values in the classification have more contributions to clinical diagnosis of disease, which means these metabolites can be used as potential biomarkers for disease diagnosis, especially in the early diagnosis. The variable importance of metabolites for each classification model is shown in Figure 4. Glyceric acid, 2, 3-dihydroxybutanoic acid, N-(oxohexyl)-glycine, and D-turanose showed the highest variable importance (higher than 0.45, Figure 4), which are more likely to be useful markers for BC diagnosis.

Glyceric acid is an important intermediate in the lipid metabolism, which can be produced by the oxidation of fatty acids and the hydrolysis of phosphoglyceric acids (such as 2-phosphoglyceric acid, 3-phosphoglyceric acid, and 1, 3-bisphosphoglyceric acid). Phosphoglyceric acids are important intermediate products of the tricarboxylic acid cycle in the organisms and directly involved in the metabolism and transformation of energy, such as 1, 3-bisphosphoglyceric acid, which is a high-energy phosphate compound in vivo and can produce one molecule of ATP to the living body under the catalysis of the phosphoglycerate enzyme.

2, 3-Dihydroxybutanoic acid is related to the metabolic pathway of L-threonine and generated by the metabolites of L-threonine, which is a ketogenic amino acid, and its metabolites can directly join in the energy metabolism.

N-(oxohexyl)-glycine is one of the acyl amino acids, exactly acyl glycine, in organisms and usually produced in the metabolic process of fatty acids with very small quantity. Acyl glycine is usually produced under the catalysis of acyltransferase. The reaction is as follows: glycine + acyl-coenzyme A ⟶ acyl glycine + coenzyme A. Acetyl coenzyme A can directly provide a molecular dicarbon compound for the tricarboxylic acid cycle. Furthermore, the combination of oxaloacetate and acetyl coenzyme A is believed to be the initial step in the citric acid cycle. As a result, the abnormalities of the acyl glycine metabolism may affect the energy metabolism of cells and form the specific metabolic pathways of tumor.

**Figure 5:**
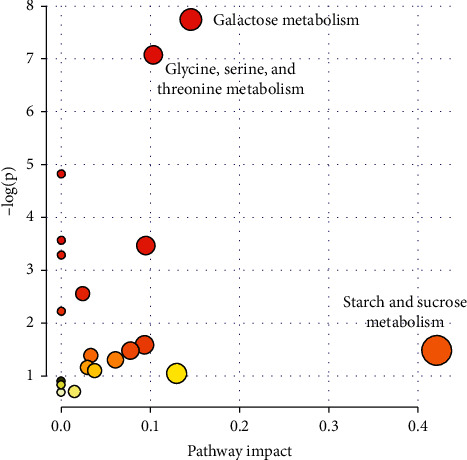
Pathway analysis based on significant metabolites.

All the abovementioned results indicated that the metabolic pathways of lipid and some amino acids changed significantly as the bladder tumor grew. Thus, based on these metabolites, the pathway analysis was implemented by using Metaboanalyte software. The pathway analysis results showed that starch and sucrose metabolism, glycine, serine, and threonine metabolism, and galactose metabolism have strong relationships with selected metabolites (shown in Figure 5).

## 4. Conclusions

Metabolomics is an effective approach to discover biomarkers by analyzing global changes in the metabolic profiles. To collect early-stage bladder cancer (BC) samples and follow-up the BC progress, the rat bladder cancer model was established by BBN inducing in this study. The metabolites in rat urine were detected with GC-MS and analyzed with random forests algorithm to distinguish the early, middle, and advanced stage of the bladder cancer group and healthy group. The results showed that urinary levels of some metabolites had a significant difference between the cancer group and the healthy group and advanced-stage group and the other two stage groups, which suggested that the growth of bladder tumor might result in the abnormality of the metabolism of lipids and some amino acids. Furthermore, glyceric acid, 2, 3-dihydroxybutanoic acid, N-(oxohexyl)-glycine, and D-turanose with the highest variable importance might be the potential markers of bladder cancer, and their metabolic pathways were studied. Yet, the data reported here are preliminary and need to be confirmed by large scale of samples. Further studies should be required to value the significance of the four compounds as the potential marker in human urine for bladder cancer.

## Figures and Tables

**Table 1:** Qualitative and quantitative results of 41 common chromatographic peaks in urine samples.

No.	*t*_*r*_^*a*^ (min)	Endogenous metabolites	Healthy group (*A*_x_/*A*_i_, *n* = 45)	Early stage (*A*_x_/*A*_i_, *n* = 45)	Medium stage (*A*_x_/*A*_i_, *n* = 45)	Advanced stage (*A*_x_/*A*_i_, *n* = 45)
1	5.922	Aminoethane	0.2456 ± 0.0705	0.1782 ± 0.0352	0.1905 ± 0.0567	0.1958 ± 0.0551
2	6.593	Ethylene glycol	0.0182 ± 0.0020	0.0373 ± 0.0107	0.0530 ± 0.0428	0.0746 ± 0.0626
3	6.840	N, N-diethylacetamide	0.0657 ± 0.0087	0.0584 ± 0.0099	0.0476 ± 0.0202	0.0557 ± 0.0107
4	7.716	Lactic acid	0.0872 ± 0.0374	0.0765 ± 0.02934	0.0952 ± 0.0592	0.1482 ± 0.2155
5	7.934	Acetic acid	0.0856 ± 0.0333	0.0341 ± 0.0254	0.0229 ± 0.0140	0.0412 ± 0.0203
6	10.010	Phosphate	2.1278 ± 0.9173	2.7785 ± 1.4037	1.4730 ± 0.7381	1.3767 ± 0.9361
7	10.200	l-threonine	0.0173 ± 0.0098	0.0052 ± 0.0029	0.0108 ± 0.0068	0.0096 ± 0.0065
8	10.297	Phenylacetic acid	0.0047 ± 0.0023	0.0183 ± 0.0054	0.0159 ± 0.0103	0.0147 ± 0.0097
9	10.382	Succinic acid	0.0311 ± 0.0129	0.0117 ± 0.0084	0.0098 ± 0.0031	0.0119 ± 0.0086
10	10.447	1, 2-Hydroquinone	0.0120 ± 0.0072	0.0103 ± 0.0073	0.0078 ± 0.0047	0.0067 ± 0.0039
11	10.503	Glyceric acid	0.0961 ± 0.0266	0.0250 ± 0.0120	0.0400 ± 0.0232	0.0183 ± 0.0087
12	10.723	(R^*∗*^, R^*∗*^)-2, 3-dihydroxybutanoic acid	0.0167 ± 0.0053	0.0045 ± 0.0013	0.0037 ± 0.0014	0.0053 ± 0.0029
13	11.357	2, 4-Dihyoxybutanoic acid	0.0147 ± 0.0051	0.0131 ± 0.0041	0.0155 ± 0.0080	0.0166 ± 0.0047
14	11.583	(R^*∗*^, S^*∗*^)-3, 4-dihydroxybutanoic acid	0.0304 ± 0.0098	0.0161 ± 0.0053	0.0132 ± 0.0064	0.0178 ± 0.0107
15	11.797	N-(1-oxobutyl)- glycine	0.0653 ± 0.0244	0.0191 ± 0.0096	0.0319 ± 0.0186	0.0274 ± 0.0151
16	12.341	Isovaleroglycine	0.0356 ± 0.0134	0.0134 ± 0.0041	0.0160 ± 0.0079	0.0107 ± 0.0073
17	12.483	D-threitol	0.0714 ± 0.0273	0.0665 ± 0.0260	0.0290 ± 0.0130	0.0251 ± 0.0151
18	12.645	N-crotonyl glycine	0.0240 ± 0.0146	0.328 ± 0.0074	0.0207 ± 0.0129	0.0148 ± 0.0099
19	12.973, 13.203	2, 3, 4-Trihydroxybutyrate	0.1276 ± 0.0162	0.0475 ± 0.0244	0.0631 ± 0.0343	0.0412 ± 0.0250
20	14.530	N-(1-oxohexyl)-glycine	0.0960 ± 0.0319	0.0112 ± 0.039	0.0421 ± 0.0273	0.0232 ± 0.0081
21	14.580	3-Hydroxyphenylacetic acid	0.0326 ± 0.0100	0.0122 ± 0.0910	0.0140 ± 0.0081	0.0134 ± 0.0088
22	14.713	D-xylose	0.0408 ± 0.0150	0.0225 ± 0.0206	0.0182 ± 0.0044	0.0193 ± 0.0053
23	14.823, 15.057	D-ribose	0.0926 ± 0.0370	0.0682 ± 0.0340	0.0252 ± 0.0142	0.0250 ± 0.0179
24	15.509, 15.733	Arabitol	0.0287 ± 0.0164	0.0252 ± 0.0168	0.0283 ± 0.0179	0.0278 ± 0.0215
25	16.023	6-Deoxy-D-galactose	0.0336 ± 0.0083	0.0370 ± 0.0064	0.0177 ± 0.0100	0.0149 ± 0.0104
26	16.087	Mannonic acid	0.0505 ± 0.0177	0.0419 ± 0.0168	0.0211 ± 0.0143	0.0168 ± 0.0138
27	16.200	cis-Aconitic acid	0.0535 ± 0.0288	0.0524 ± 0.0209	0.0105 ± 0.0079	0.0168 ± 0.0147
28	16.357	Phosphoric acid	0.0414 ± 0.0202	0.0463 ± 0.0177	0.0230 ± 0.0141	0.0212 ± 0.0168
29	17.177	Isocitric acid	0.0348 ± 0.0121	0.0410 ± 0.0157	0.0140 ± 0.0093	0.0248 ± 0.0138
30	17.563	Hippuric acid	0.0470 ± 0.0126	0.0201 ± 0.0111	0.0180 ± 0.0074	0.0156 ± 0.0096
31	17.850, 17.960	D-fructose	0.0512 ± 0.0286	0.0728 ± 0.0106	0.0371 ± 0.0145	0.0480 ± 0.0131
32	18.087	N-phenyl glycine	0.0596 ± 0.0214	0.0363 ± 0.0194	0.0455 ± 0.0272	0.0389 ± 0.0287
33	18.197, 18.147	D-glucose	0.3785 ± 0.1618	0.2254 ± 0.1328	0.1741 ± 0.0654	0.1859 ± 0.0736
34	18.507	Altronic acid	0.0302 ± 0.0069	0.0164 ± 0.0106	0.0185 ± 0.0100	0.0102 ± 0.0074
35	18.577, 18.650	D-sorbitol	0.0896 ± 0.0269	0.0623 ± 0.0171	0.0254 ± 0.0187	0.0300 ± 0.0275
36	18.983, 19.533	Galactonic acid	0.0613 ± 0.0282	0.0387 ± 0.0186	0.0617 ± 0.0328	0.0441 ± 0.0351
37	19.990	Palmitic acid	0.0084 ± 0.0009	0.0085 ± 0.0047	0.0067 ± 0.0017	0.0071 ± 0.0025
38	20.403	Myo-inositol	0.0347 ± 0.0228	0.0089 ± 0.0033	0.0097 ± 0.0037	0.0134 ± 0.0129
39	25.465	D-turanose	0.0216 ± 0.0138	0.0145 ± 0.0118	0.0197 ± 0.0090	0.0510 ± 0.0099
40	25.653, 25.783	D- (+)-lactose monohydrate	1.0400 ± 0.3349	0.9997 ± 0.2385	0.7475 ± 0.2366	0.6559 ± 0.3286
41	25.927	Lactose	0.0142 ± 0.0043	0.0231 ± 0.0039	0.0143 ± 0.0075	0.0190 ± 0.0163

No: the serial number of the common peak; *t*_*r*_^*a*^: retention time. *A*_x_/*A*_i_: the ratio of the peak area of the analyte to that of the internal standard.

**Table 2:** Classification group for three models.

Results	Sensitivity (%)	Specificity (%)	Accuracy (%)	MCC	AUC
Healthy control vs. early- and middle-stage group	83.33	98.80	96.06	0.8609	0.9775
Healthy control vs. advanced-stage group	97.22	97.60	97.54	0.9194	0.9868
Early- and middle-stage groups vs. advanced stage group	80.56	97.01	94.09	0.7909	0.9546

MCC: Matthews correlation coefficient, AUC: area under curve.

## Data Availability

The figure data and related data used to support the findings of this study are included within the article.

## Supplementary Materials

Supplementary Materials Figure S1: the total ion chromatograms (TIC) of samples with different extraction solvents. Figure S2: the total ion chromatograms (TIC) of samples with different dosages of urease. Figure S3: the total ion chromatograms (TIC) of samples with different decomposition temperature. Figure S4: the total ion chromatograms (TIC) of samples with different decomposition time.
