# Supplementary material for: A Urine Metabonomics Study of Rat Bladder Cancer by Combining Gas Chromatography-Mass Spectrometry with Random Forest Algorithm
Source: Int J Anal Chem. 2020 Sep 21;2020:8839215. doi: 10.1155/2020/8839215 (PMC7525317; doi:10.1155/2020/8839215)
Supplement: Supplementary Materials — Figure S1: the total ion chromatograms (TIC) of samples with different extraction solvents. Figure S2: the total ion chromatograms (TIC) of samples with different dosages of urease. Figure S3: the total ion chromatograms (TIC) of samples with different decomposition temperature. Figure S4: the total ion chromatograms (TIC) of samples with different decomposition time. [file 8839215.f1.docx]

Supplementary Materials

**A Urine Metabonomics Study of Rat Bladder Cancer by Combining Gas Chromatography-Mass Spectrometry with Random Forest Algorithm**

Mengchan Fang 1, Fan Liu 2，Lingling Huang 1, Liqing Wu 3, Lan Guo1, 2*, Yiqun Wan 1, 2*


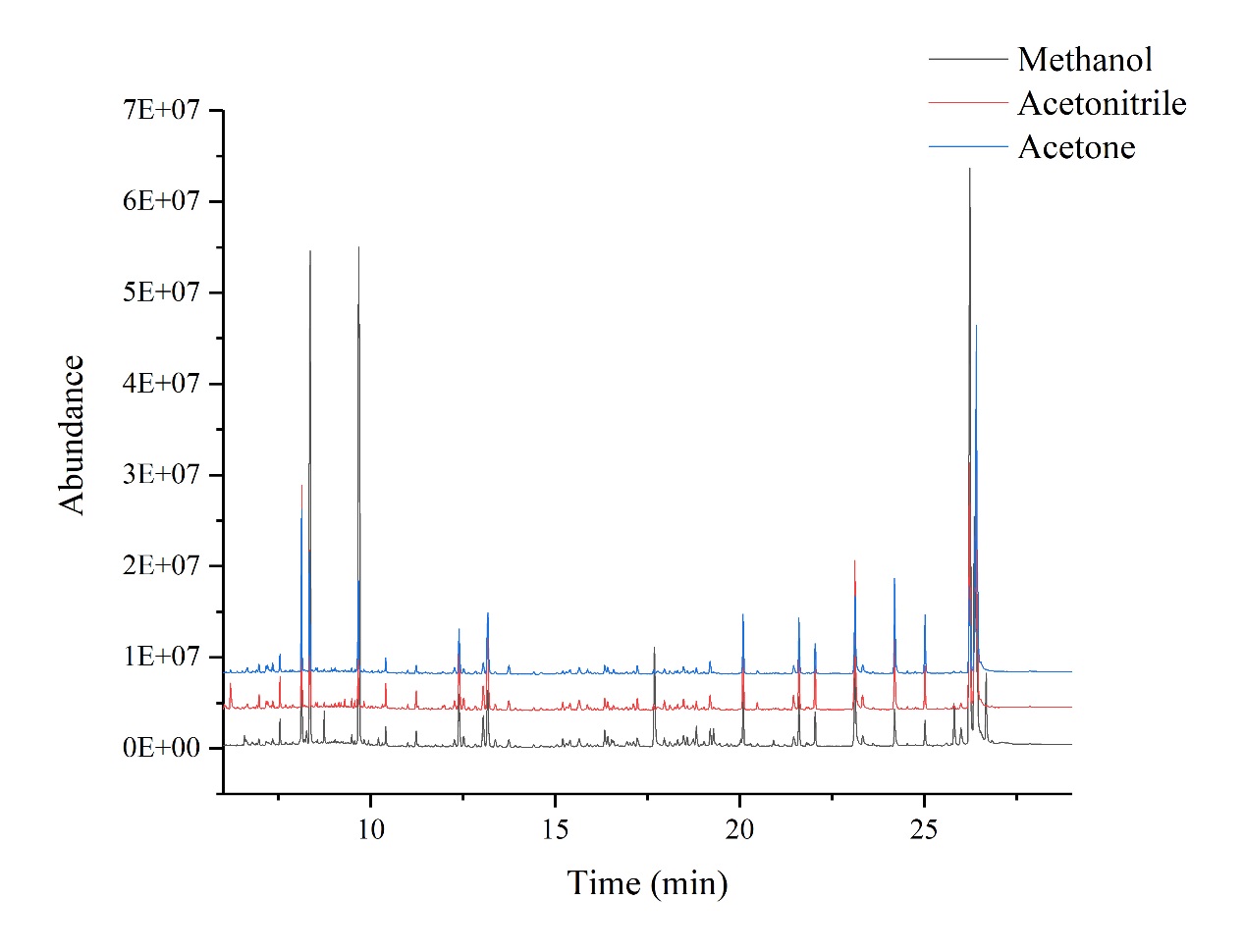


Fig. S1 The total ion chromatograms (TIC) of samples with different extraction solvents.


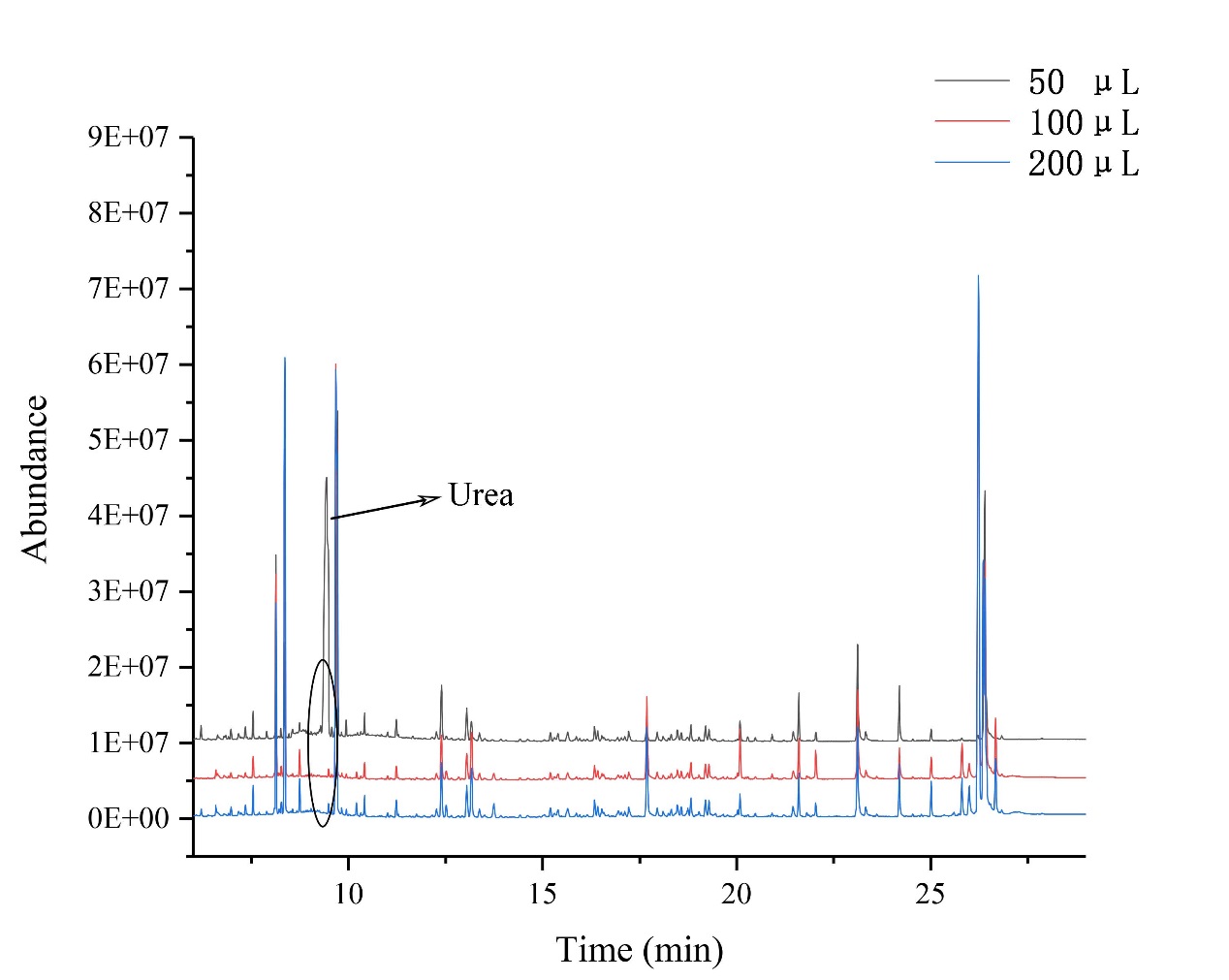


Fig. S2 The total ion chromatograms (TIC) of samples with different dosages of urease.


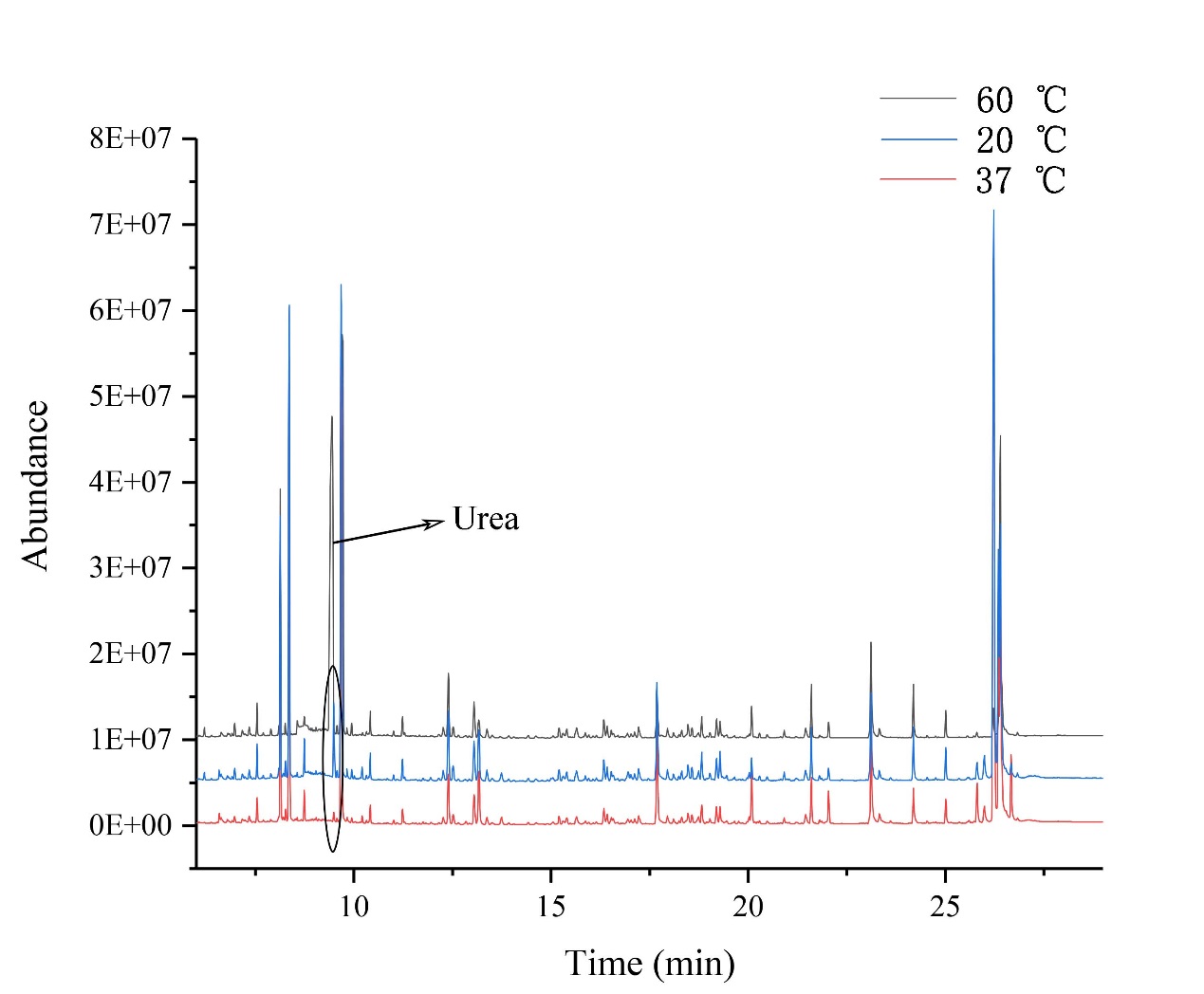


Fig. S3 The total ion chromatograms (TIC) of samples with different decomposition temperature.


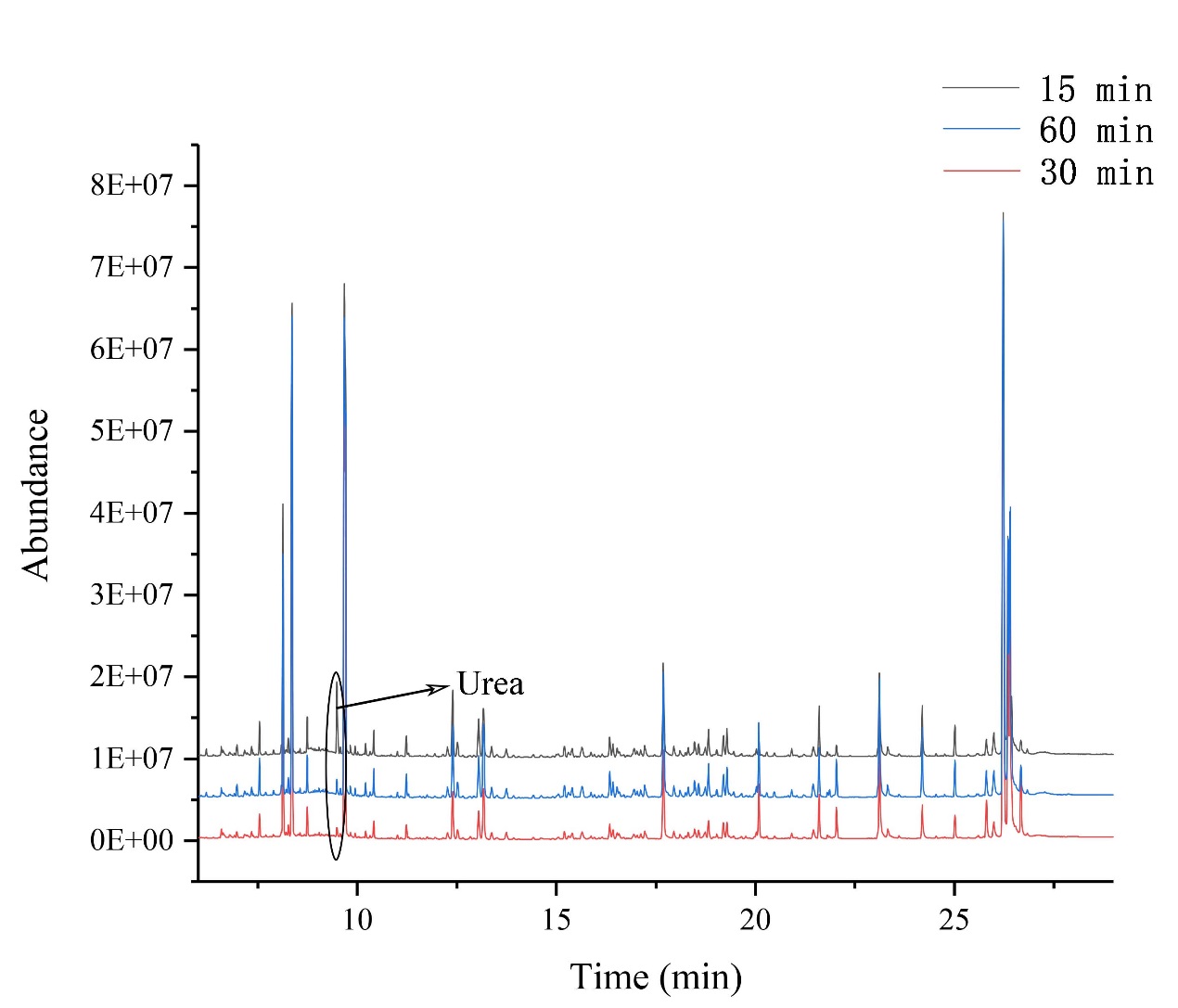


Fig. S4 The total ion chromatograms (TIC) of samples with different decomposition time.
